# Intelligence

**Published:** 1827-10

**Authors:** 


					INTELLIGENCE.
Tn monthly report of prevalent diseases.
jnnjJ3 .'ast ",0nth lias not been remarkable for any peculiarity of disease, of
CU-'H interest to be recorded.
Coll(Se of Physicians -In our last Number, we Rave insertion to a letter
,'01n " A Licentiate," in which he strongly urges the imprudence of the College
?f Physicians, in interfering with Dr. Harrison. We have already (Number
'or July) detailed our reasons for thinking, on the contrary, that the College
Vere imperatively called upon to take some steps in the business ; and we shall
Ao. 314.? A'o, i(j( New Series. ^ ^
374 ? INTELLIGENCE.
now only remark?that the gauntlet having been thrown down to them in *',c
most taunting manner, before, the profession and the public, to decline tl'e
contest would be attributed?not to dignified indifference, but to pusillanimity'
or to the consciousness of having assumed a jurisdiction in which they are not
borne out by their Charter.
It was our intention to have commented on the letter of our correspondent)
whose principal object seems to have been to dissuade the College from inter-
fering; but, from the Correspondence between the Censors and Dr. Harrison*
which we now publish, it appears that the matter has gone too far to admit of
the College following the Licentiate's advice, even should they be so disposed'
The College having " admonished" Dr. Harrison, they are bound, injustice
to themselves, and for the sake of consistency, to prosecute?if he continue
to practise, and if they obtain legal evidence of his doing so: otherwise, it would
indeed reduce their admonition to what Dr. Harrison insinuates it is?a nie'e
Brutum fulmen.
In our view of the case, however, both the above conditions are rcquisite
to enable the College to bring the matter to issue; and it is proper to keeP
this in mind. Suppose the College could prove that Dr. Harrison had practise
anteriorly to his being served with a notice to desist from doing so, we appreben
tliat they could not found an action thereon. Suppose, again, that Dr. Harriso'j
should continue to practise, yet, unless legal evidence could be obtained o
the fact, the po\ver of the College would still be unavailing. This evidence>
we believe to consist in prescriptions signed with the initials of the physician
?at least, this is the kind of evidence which has been used 011 former occa-
sions. Now, as it is obvious that any one who is desirous of so doing cal1
prevent the possibility of such testimony against him, so the College may
unable to bring the question, which now excites so much interest, to the de-
cision of a Court of Law, without either their wanting the will to do so, or the
authority of their Charter being invalidated. Failure from the absence ot
sufficient proof, is very different from failure/ because there is no penalty at-
tached to the offence when proved.
In making these remarks, we would by 110 means wish it to be supposed
that we take on ourselves the office of advocates for the College of Physicians:
but, perceiving that there is much misapprehension on the subject, we think
it right to state what appears to us to be the fair view of the case, believing
as we do, that, according to the Charter of Henry the VHIth, confirmed by
Parliament,* the College of Physicians have the power to levy a fine of
per month on every physician who shall continue to practise in London an('
its vicinity after he has been admonished to desist,?provided they can brio?
evidence of his doing so.
There is another point likewise oil which we beg not to be misunderstood*
In alluding to the facility with which the necessary evidence can be withheld*
we do not mean to insinuate that Dr. Harrison has any intention of adopt'11?
this subterfuge : indeed, he has declared his earnest desire 11 to come at once to
the points at issue;" and, to throw any difficulties in thtf-way of evidence that
he does actually practise, would not only prevent this, but at the same time
reduce all that lie lias done to mere vapouring and bravado. Let Dr. Harriso11
put the College in possession of the requisite evidence as to the f"ct ot
practising, and then it will only remain to decide the question as to the rigM
of doing so.
* The Charter of King James was not confirmed by Parliament.
Di\ Harrison and the College of Physicians. 375
av?idcdaVe '1Gart' lcma'ked, that Dr. Harrison, unlike other physicians,
l)r?Cee(J fPUUing '''S nan,e uPon liis door, and this many have supposed to
Percei '?ni ^eai t'le College; since these discussions began, however, we
l'lat ^'e Sro"uds of such an idea have been removed, and Doctok
avoid IS?N nou aPPears at full length. This looks as if he were anxious lo
tllat thing that can be construed into evasion ; we therefore anticipate
Pr?fess'Wl" a(*?'>t the ''int which we have thrown out, and tints convince the
Co?eo that he is in earnest. It would then certainly not be his fault if the
<<jt w.? echned bringing the matter to issue: but, as the Censors state that
c'?ubt >C l'1G the said College to proceed" against him, we have" no
Court ofat t,1C ^uest'on W0l,ld immediately be brought to the decision of a
obtain? ' au ?hject which Dr. Harrison professes himself so anxious to
pMidaice between Dr. Harrison and the Censoks of the College of
Physicians.
No. I.
^?Py of the Censors'first Communication to Dr. Harrison.
M'e, Ule f, JnhJ 6t'b 1827.
'"fori ? ensors l'ie Royal College of Physicians, London, having received
seven 1011 "lat you are practising physic within the city of London, and
tiiyou ',CS,|?.f ,he same, do hereby admonish you to desist from so doing, un-
real 0p ia 'lave been duly examined and licensed thereto, under the common
Proceed' ^ ^?"eSe > otlierwise it will be the duty of the said College to
agamst you for the recovery of the penalties thereby incurred.
WILLIAM LAM BE,
J. COPE,
H.-H. SOUTHEY,
Collet r j-, CORNWALLIS HEWETT7
A 8e "f Physicians, Pall Mall East.
he l10|l?a' ^ ^or examining persons who have the requisite qualification, will
y^en at the College on next Friday, 13th July, 1827.
0 Dr. Edward Harrison, Hollis-street.
No. II.
1"J of l3r. Harrison's first Answer to the Censors' Communication.
q Holies-street; ?th July, 18-27".
purn .enie,V?I had last night the honour of receiving a communication,
siciansr lnS to he signed by yon, as the Censors of the Royal College of Pliy-
l)riictiS' ^?n<'on' wherein you are pleased to admonish me to desist from
ullt|1 jSll,S Physic within the city of London, and seven miles of the same,
1JJon 'Iave been duly examined and licensed thereto, under the coiu-
of i|,gta'.0^ l'le sa'd College; and alleging that, otherwise it will be the duty
tlicr i Sa'^ ^?"eSe to proceed against me for the recovery of the penalties
}^y incurred.
point 01U ^ ailswer the above communication, will you have the goodness to
y0lI aj""j me "ie '"uithorities under which you act, and the penalties to which
I have the honour (o be, Gentlemen,
Your very obedient humble servant,
To n? , , EDWARD HARRISON.
s. iMinbe, Cope, Southey, and Heivelt,
?yul College of Physicians, London.
376 INTELLIGENCE.
No. lit.
2, King's-road, Bedford-row ; 9/A ^~'o0r
Sir,?Being senior Censor of the College of Physicians, I opene j
letter of the 7th instant, which I shall lay before the Board at then ^
meeting. I think it right to inform you, that such meeting is appointe ^
Friday next, at three o'clock, where you may appear, if you think proper > ^
obtain whatever information the Board may think it their duty to com
cate to you. I am, sir, your obedient servant, -
WILLIAM LAMB*"
Dr. Harrison, Holies-street, Cuvendish-square.
No. IV.
Holles-streel; July 11th, ^
Sir,?I beg to acknowledge the receipt of your letter of Monday last, 3
I have only to repeat the request made by my former letter.
I have the honour to be, sir,
Your very obedient humble servant,
EDWARD HARRISON*
Dr. Lambe, 2, King's-road, Bedford-row.
No. V.
Copy of Censors' second Communication to Dr. Harrison.
College of Physicians, July 19th, l827'
Sir,? We, the Censors of the Royal College of Physicians, London, hav^ ^
taken into consideration the request made in your letter of the 7th Ju y> ^
be informed under what authorities we act, and what are the penalties ^
which we alluded'?have to inform you, that we act under the authouty ^
our Charter, confirmed by Parliament, 14th and 15th Henry VIII.>whic
well known, and has been repeatedly enforced.
WILLIAM LA.MBE, ,
CLEMENT HUE, (vicarius for Dr. Cop*,/
H. H. SOUTHEY,
CORNWALLIS HEWETT.
There will be a Censors' Board held at half-past four o'clock on Thins ^
next, 26th July, at which, if you think proper, you will have an opportuiJi y
appearing.
Dr. Edward Harrison, Ilolles-street.
No. VI.
Copy of Dr. Harrison's second Answer to the Censors' Communication?
Holies-street; July <23(1, 1827.
Gentlemen,?After acknowledging the favour of your letter of the 10*"
instant, informing me " that you act under the authority of your Charter?
confirmed by Parliament 14th and loth Henry VIII.," I beg leave to observe,
that I can no where find the title of " Censors of the Royal College of Fhy"
sicians" mentioned in that statute; or their right to examine graduates o
Universities recognised. And, what is no less relevant in this case, it d?c*
not appear to confer the power of constituting two different classes of physl"
cians, under the denomination of Fellows and Licentiates. Such is |'jC
result of a careful examination of the above-named act. But if you can |>?int
l
Dr. Harrison and the College of Physicians. 377
??t particular clauses in it by which the title and powers in question
tinctly given, I shall feel obliged by the communication.
I have the honour to be, gentlemen,
Your very obedient '^^HIUSON.
To Drs. Lambe, Cope, Soutliey, and Hewett,
College of Physicians, London.
No. VII.
Copy of Censous' third Communication to Di. H 1827.
CoI^0/PtvT"ceted y;u-letter,
We, the Censors of the College of Physicians, ^ t0 our last
tearing the date of July the 23d, 1827, anI have for examini?g all
c?mniunication, excepting that the next ens College of
Persons who have the requisite qualifications, will "be held
Physicians on the 1st of next October, at LAMBE,
v |31lV?sE hewett.
Dr. Edward Harrison, Holies-street>
Dr h No.VIII. <
arrison to the Censors of the College, (delivered August 6th.)
Q | Holies-street; August 4th, 1817.
?h, i St.1" ??*>your communication, bearing date of the 26th of
t'ons for t0 State thatI was ,et*in '?y ,ast letter to propose three ques-
s'?n> and^0"1 cons^erat'on> wit'1 the view of encouraging amicable discus-
As Biy c ?^.Pro^u?ing> if possible, a conformity of sentiment between us.
answer j?nci''atory efforts have been frustrated by your uncompromising
ti?n ' eS to inform you, that, having bestowed no inconsiderable atten-
tat th?n ^'e const'tution of the College of Physicians, I am led to conclude
Henrv'v^"V^e^eS an(* Povvers granted to it by the statute 14th and 15th of
colie are n?tnow in existence, or at least are no longer available for
able d fr UrpOS^S" ^Pon the consideration generally of the other insurmount-
it ? lc"lties and objections to the exercise of your monopoly, I shall think
I?teli?eSSary t0 enter? ""less the paramount one can be removed.
law 'Sent and enlightened physicians, as well as gentlemen learned in the
even Cnterta*n similar opinions to my own. I have also reason to know that,
b?ast ^nonS those who formerly ranked with the highest of the Fellows, the
As IJ ,autk?rity of the College was denominated a mere " Brutum fuhnen."
cjSe o^.a n?t lately met with opposition from any of the Fellows in the exer-
them ^ Pro^ess'ona' duties, I concluded that I should be suffered;to.pursue
qUjet u'thout further molestation,?until I was roused from my deceptive
p ' an^ forced into the field by your colleague Dr. Chambers,
pi,y ? } the concurring approbation of accomplished lawyers and
Cai n lans> * thought that I could not bestow greater service upon the medi-
d'sn t? eSS'0n' to w^ich I am enthusiastically devoted, than by bringing all
the U Matters formally into open Court, under a conviction that, however
t, decided, the interests of the faculty and of the public will be
^ntia"y promoted by the investigation.
5ea?C tCd tliese motives, I have tendered to the College, for a series of
p,. ,jS' through some of its Fellows, opportunities of examining legally their
0 ensigns to interfere with me, or my practice.
378 INTELLIGENCE.
I am unwilling to suppose that the gentlemen who now compose the Royal
College of Physicians, and for whom, individually, I entertain every respect?
are less desirous than myself to come at once to the points at issue between
us, without the introduction of those foreign topics which have hitherto em*
barrassed the question, and prevented a satisfactory decision.
Acting on public grounds only, and for the advantage of our common pro-
fession, I pledge myself that, if proceedings are instituted, they shall, on "V
part, he carried on without unnecessary irritation or excitement.
In pursuance of the great objects for which I contend, I now declare that 1
do not recognise your authority as " Censors of the Royal College of Physl'
cians," and shall therefore decline your invitation to offer myself at your L1
censing Board on the 1st of next October.
I have only to add, in concluding my correspondence, that Messrs. Tennantj
Harrison, and Tennant, of Gray's Inn, are my solicitors. To them I rC^cl
you, in case of your choosing to insiitute proceedings against me. They a"'
furnished with instructions to give every facility to a legal investigation o
your assumed privileges; but they are directed neither to compromise niy
rights, nor those of my professional brethren.
I have the honour to be, gentlemen,
Your very obedient humble servant,
(Signed) EDWARD HARRISON-
For Drs. Lambe, Cope, Southey, and Ilewett, College of Physicians.
New Regulations of the Company of Apothecaries.?[It will be perceived that
the following regulations do not apply to the pupils who come to town this
autumn, and will not, in fact, be in full force till October, 18'.?8.]
The Court of Examiners chosen and appointed by the Master, Wardens
and Assistants of the Society of Apothecaries of the City of London, in p?rsU*
ance of a certain Act of Parliament, " For better Regulating the Practice o
Apothecaries throughout England and Wales," passed in the 55th year of tl'e
reign ot his Majesty King George the Third, have determined?
That every candidate for a certificate to practise as an Apothecary shall b<?
required to possess a competent knowledge of the Latin language, and to
produce testimonials of having served an apprenticeship of not less than five
years to an apothecary; of having attained the full age of twenty-one years*
and being of a good moral conduct.
N. B. Articles of apprenticeship, where sneli are in existence, will be ,c
quired; but, in case such articles have been lost, it is expected that the cau
didate shall bring forward very strong testimony to prove that lie has serve1
such an apprenticeship, as the Act of Parliament directs.
He is also required to produce certificates of having attended not less tha"
one course of Lectures on Materia Medica and Medical Botany ; one course
of Lectures on Chemistry; two courses of Lectures on Anatomy and PhySI*
ology; two courses of Lectures 011 the Theory and Practice of Medicine:
these last to be attended subsequently to the Lectures on Materia Medica,
Chemistry, and to one course, at least, of Anatomy.
N.B. No testimonial of attendance 011 Lectures on the Principles and Prac-
tice of Medicine, delivered in London, or within seven miles thereof,
render a candidate eligible for examination, unless such Lectures were giveH;
and the testimonial is signed by, a Fellow, Candidate, or Licentiate, of the
Royal College of Physicians.
A certificate of attendance for six months, at least, on the medical practice
Regulations of the Compan y of Apothecaries. 373
*01tle public Hospital or Infirmary, or for nine months at a Dispensaiy .
c'C ' a^en('ance to commence subsequently to tlie termination o tie lis
?"'se of Lectures on the Principles and Practice of Medicine.
N-B. Physicians' pupils, who intend to present themselves for examination,
J?nst appear personally at the Beadle's Office, in this Hall, and bring with
'cni the tickets authorising their attendance on such practice, as the com-
"encenient thereof will be dated from the time of such personal appearance.
l lle regulations relating to the order of succession in which the Lectures 011
,e Practice of Medicine, and the medical practice of an Hospital or Dispen-
are to be attended, are designed to apply to those students only who
? 'aH commence their attendance on Lectures on or after the 1st ot February,
J'-8; and all such persons are particularly requested to take notice that, un-
*ss they 5hau have strictly complied with such order of succession, they will
?t be admitted to an examination. . .
In addition to the course of study above required, and which is mdispensa-
"y necessary, the candidates are earnestly recommended to attend one or
'?ore courses of Lectures on Midwifery, and the Diseases ot Women am
'"tdren; on the latter of which subjects, as an important part of medica
^t'ce, they will he examined.
I ho ri
'X'lj0 p * ""** VAUIIJIIICUi
as fnii ?Urt 'IUve determined, that (he examination of the candidate shall be
,0|lows:__
a*id nh'1 !lans,at?n? grammatically parts of the Pharmacopoeia Londinensis,
s,10jraians;prescri?tions'
'edge of t 1^,t anse as t0 the candidate's possessing a competent know-
S0||ie oi atin' ^'e w'" re(lu'red to translate a passage, or passages, from
easier Latin authors.
iiecessit iG ^ourt are anxious to impress upon candidates a conviction of the
painf,,! -3 knowledge of the Latin language, because they have had the
their (je^n.^ ln,posed upon them of rejecting several persons, entirely from
2# T Clei!cy this important pre-requisite of a medical education.
^naton Iemistl'y* 3- In the Materia Medica and Medical Botany, 4. In
my a"d Physiology. 5. In the Practice of Medicine.
to ille ' "f ^ie Rhododendron Chrysanthum.?Sir, It has frequently occurred
tlie yelfo3^' t'lrouS'1 the medium of your Journal, where the dried foliage of
?nulllei.?W'flowered Rhododendron could be procured, as it has long been
''ave er)3/6'' l'le ^ater'a Medica list of the Edinburgh Pharmacopoeia. I
lately Jla eaV0HretI ^or several years past to purchase this article, and till very
Vxei'e 01 >VC n0t ^een successful. The leaves of Rhododendron Caucasicum
*'le pro (C S?'C' '? me *or t'i0se R* Chrysanthum. Much has been said about
s'?n wii| rt'eS "1's P'ant> and 'ts beneficial effects in gout. The profes-
n?*> and'l^ ''avc an opportunity of ascertaining whether it really is useful or
Jlr j> 1 think it but right to inform them where it can be now procured.
c?iside jG1' *'le n,edicinal herb vender, of Covent Garden, has imported a
water at 2 eo(^liant'ty from Siberia. Its active principles are given out to
biting it ?* ^a'lrenheit. Infusion appears to be the best mode of exhi-
A uine ' aS ^ coction some of the more volatile properties are dissipated.
Plant G a'}^ a t'nct,Ire may be formed from them. Tlie proportions of the
atto 'Je regulated by experience; and my only object is to draw the
1011 of the profession to it.
c>o r . I am, .Sir, your obedient servant, J. FROST.
> rt(lge-streel, Blackfriars; August 1827.
METEOROLOGICAL JOURNAL,
By Messrs..Harms and Co. Mathematical Instrument Makers, 50, HighHolborn.
20
21
22
23
24
25
20
27
28
2!)
SO
31
Sept
2
3
4
5
6
7
8
a
10
r.
12
13
14
15
16
17
18
19
.13
o
Thermom.
Barometer.
29.92
29.97
30.09
30.20
30.14
30.00
30.07
30.17
30.17
30.26
30.17
30.17
30.23
30.22
30.18
30.20
30.11
20.1C
30.16
30.13
29.90
29.76
29.72
29.61
29.77
30.09
30.(4
30.18
30.21
30.13
30.04
29.92
30.01
30.15
30.21
30.01
30.06
30.10
30.19
30.21
30.23
30.05
30.22
30.24
30.20
30.20
30.15
30.17
30.18
30.14
30.07
29.82
29.76
29.62
29.68
29.98
30.08
30.15
30.20
30.17
30.07
30.03
72
Winds.
NNE
i E
NE
NNEv
! N
NNEv
NNEv
NE
. NW
NNE
VVNW
NE
i
! E
ENE
ENE
ENE
ENE
ENE
j ENE
! NE
! S
WNW
s\v
ssvv
I W
! w
! N
1 ENE
ENE
! SE
NW
NNW
NE
E
NE
NNW
N
N
NNW
NNE
N
NW
ESE
E
E
E
E
ENE
ENE
E
WSW
WNW
W
WSW
WNW
WN W
NNW
NNE
SE
N
NW
Atmospheric Variatio n.
9 a.m.
Fair
Fine
Fair
Overca.
| Fair
iCloudy
jSleet
Cloudy
I Fair
jClouily
| Faii-
Cloudy
Faii-
Cloudy
Fair
I Rain
Cloudv
SI. Rain
Cloudy
Faii-
Cloudy
Fair
jCloudy
2 p.m.
Fine
Fair
SI. Rain
Fine
Fair
Fine
Cloudy
Fair
Fine
Faii-
Cloudy
10 p.m
Cloudy
Fine
Cloudy
Fine
Fair
Cloudy
Fair
Fine
Cloudy
Ruin
Cloudy
Rain
Fair
Rain
Fair jFair
? ICloudy
[Fair
? cloudy
Fine Fair
Fair Rain
Fair |  iCloudy
The quantity of Rain fallen in the month of August, was 1 inch anu 8.100tlia.

				

